# The Application Value of Brain Natriuretic Peptide in the Prognostic Evaluation of Patients With Chronic Left Heart Failure

**DOI:** 10.1155/cdr/9353377

**Published:** 2025-08-16

**Authors:** Congling Xiang, Weier Zhou

**Affiliations:** ^1^Department of Cardiovascular Medicine, The People's Hospital of Xinchang County, Xinchang, China; ^2^Department of Geriatric Cardiology, Traditional Chinese Medicine Hospital of Zhuji, Zhuji, China

**Keywords:** brain natriuretic peptide, chronic left heart failure, evaluation value, prognosis

## Abstract

This study explores the predictive utility of brain natriuretic peptide (BNP) levels in assessing outcomes for patients with chronic left heart failure (LHF). A cohort of 59 patients diagnosed with chronic LHF was compared to 59 healthy controls. BNP levels, alongside other cardiac function parameters, such as left ventricular ejection fraction (LVEF) and left ventricular end-systolic dimension (LVESD), were evaluated. Results revealed that BNP levels were positively correlated with worsening cardiac function and negatively correlated with LVEF. In individuals with obesity or Type 2 diabetes mellitus, BNP levels were unexpectedly lower, whereas patients with chronic kidney disease or atrial fibrillation exhibited elevated BNP levels. A BNP cut-off value of 98.9 was identified, demonstrating high diagnostic sensitivity (91.53%), specificity (83.05%), and accuracy (88.14%). The area under the ROC curve (AUC) indicated strong diagnostic performance for BNP in predicting adverse cardiovascular events. The findings underscore BNP's value not only as a biomarker for diagnosing LHF but also in prognostication and tailoring patient-specific interventions. Future studies should validate these findings across diverse populations to enhance clinical application.

**Trial Registration:** Chinese Clinical Trial Registry identifier: ChiCTR2500101966

## 1. Introduction

Brain natriuretic peptide (BNP) has special biological properties and plays an important role in the clinical diagnosis of cardiovascular diseases, which is currently receiving increasing clinical attention, especially in the diagnosis of CHF disease, which is unanimously recognized by the medical community [1]. CHF is a complex heart disease characterized by a serious stage of the disease, and the disease's mortality and morbidity are high. Data show that with increasing age, the overall disease's morbidity and mortality are increasing, so early diagnosis of the disease is of great importance [2].

A definitive gold standard for diagnosing CHF disease is lacking, and clinical assessments typically rely on the associated signs and symptoms. However, due to the low specificity of these clinical indicators, accurately determining the impact of factors such as obesity, age, and comorbidities on the clinical diagnosis becomes challenging. This complexity underscores the pressing necessity for a diagnostic method that is practical, convenient, and cost-effective [3, 4]. Cardiomyocytes are significantly stretched, and plasma BNP release increases, and this index has a better effect on venous and arterial dilation and inhibition of cardiac sympathetic nervous system activity. The plasma BNP index test is easy to operate and can be used as an important plasma marker for disease judgment, which can provide good guidance for diagnosis and prognosis of disease. This research is the initial investigation to demonstrate a meaningful relationship between BNP levels and the indexes of left ventricular end-systolic dimension (LVESD) and left ventricular ejection fraction (LVEF) in individuals suffering from left heart failure (LHF). This emphasizes BNP's potential role as a prognostic indicator for this patient population. These results pave the way for the development of focused therapeutic approaches that leverage BNP levels.

## 2. Materials and Methods

### 2.1. General Materials

In the LHF group (*n* = 59), 35 participants were male and 24 were female, with an average age of 53.41 ± 5.27 years. The patients in the LHF group were enrolled from August 2023 to July 2024. Conversely, in the healthy control group (*n* = 59), there were 37 males and 22 females who had a mean age of 53.42 ± 5.21 years. The control group consisted of individuals who underwent routine health check-ups during the same period (July 2022 to August 2024), and they were selected based on the absence of chronic diseases, including LHF.

Patient inclusion criteria included (1) patients meeting the criteria of chronic LHF diagnosis, defined by symptoms lasting over 6 months, NYHA Classes III–IV, and LVEF < 40% [5]; (2) patients with symptoms such as significant myocardial ischemia; (3) patients with interhospital revascularization; (4) patients with TIMI flow Grade 0 on coronary angiography; and (5) patients with dynamic evolution of myocardial markers or exceeding normal values.

Patient exclusion criteria included (1) patients with concomitant diseases such as tumor, myocardial infarction, or pulmonary infection 6 months before admission; (2) patients with chronic diseases such as abnormal liver and kidney function; (3) patients with immune dysfunction or infectious diseases; (4) patients who could not cooperate with prognostic follow-up; (5) patients with incomplete information; and (6) patients who experienced acute exacerbation of LHF during the study period, including acute coronary syndrome (ACS), pulmonary infection, or other acute causes of heart failure deterioration.

The study was approved by the Ethics Committee of Xinchang County People's Hospital (No. 2024-K-028-01) and was conducted according to the Declaration of Helsinki. Detailed information regarding the study can be accessed via the following website: https://www.chictr.org.cn/. All participants were informed in detail about this research and gave their written consent.

### 2.2. Blood Sample Collection

Blood samples were collected from all hospitalized patients during their admission. Specifically, for patients in the chronic LHF group, the reasons for admission were carefully reviewed to ensure that none had acute decompensated heart failure (ADHF) or ACS at the time of hospitalization. Only patients in a stable phase of chronic LHF, without acute exacerbation or acute events, were included in the study. For hemodynamically unstable patients, blood samples were drawn immediately upon arrival, prior to any major therapeutic interventions. For patients who had been stabilized during their hospital stay, additional blood samples were collected at the time of discharge, after clinical stabilization was achieved. This ensures that the BNP levels measured reflect the chronic LHF state and are not influenced by acute decompensated episodes or ACS.

### 2.3. Observation Indexes

#### 2.3.1. Examination of the Cardiographic Metrics and Overall Characteristics of the Two Groups

Patient information, including gender, age, diastolic blood pressure, and systolic blood pressure, was gathered. The automatic cardiovascular function tester (ZXG-F) was employed under ultrasound guidance to assess the LVESD.

#### 2.3.2. Left Ventricular Diastolic Internal Diameter (LVEDD)

Additionally, indexes such as the LVEF and left ventricular weight index (LVMI) were measured using the same automatic cardiovascular function tester (ZXG-F) under ultrasound assistance.

#### 2.3.3. BNP Levels at Different Cardiac Function States

BNP concentrations were recorded for NYHA Class II, Class III, and Class IV, respectively.

#### 2.3.4. Correlation Between BNP Indexes and Cardiac Function Indexes

Spearman's method was applied for correlation analysis.

#### 2.3.5. Diagnostic Value Corresponding to Different BNP Cut-Off Values [6]

Patients were followed up continuously for 3 months, during which plasma BNP corresponding to adverse event prediction ROC curves was plotted, and plasma BNP diagnostic specificity, accuracy, and sensitivity were calculated.

### 2.4. Statistical Methods

#### 2.4.1. Statistical Analysis

The analysis of data was conducted utilizing the SPSS22.0 statistical software. For datasets that followed a normal distribution, count data were represented through composition ratios and rates. The chi-square test was employed to evaluate intergroup variability. Measurement data were presented as mean ± standard deviation, while correlation analysis utilized the Spearman method. Additionally, the ROC curve method was implemented to assess the prognostic predictive value of BNP concentration. A *p* value of less than 0.05 was considered statistically significant. GraphPad Prism 8 was used for graphical representation of the results.

#### 2.4.2. Internal Validation and Sensitivity Analysis

To confirm the reliability of our results, we conducted an internal validation employing bootstrap resampling methods. This approach enabled us to evaluate the consistency of our BNP cut-off values along with their related specificity, sensitivity, and accuracy. Furthermore, we performed cross-validation of our findings across various patient groups, including individuals with comorbidities like obesity, Type 2 diabetes mellitus (T2DM), and chronic kidney disease (CKD), to validate the applicability of our conclusions.

## 3. Results

### 3.1. Analysis of Cardiac Indexes and General Data Characteristics of the Two Groups

The analysis conducted between the LHF group and the healthy control cohort uncovered several important distinctions. Although factors such as gender, age, and diastolic blood pressure were comparable across both groups, the LHF group exhibited a markedly elevated systolic blood pressure (*p* < 0.001). Evaluations of cardiac function indicators demonstrated that LVEF was significantly reduced, while LVESD, LAD, LVEDD, heart rate, and LVMI were considerably increased in the LHF group (*p* < 0.001 for each measure). Levels of blood creatinine, CRP, and BNP were also significantly higher in the LHF group in relation to the controls (*p* < 0.001). Moreover, there was a greater prevalence of obesity, Type 2 diabetes, and CKD in the LHF group (*p* < 0.05 for all), although the rise in atrial fibrillation did not reach statistical significance (*p* = 0.077). These findings can be referenced in Table 1.

### 3.2. BNP Levels in Different Cardiac Function States

**Figure 1:**
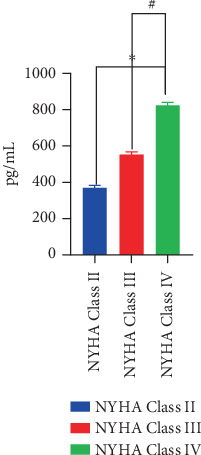
BNP levels at different states of cardiac function.

BNP concentration increased with the increase of cardiac function grade, and there was a positive correlation between the two, as shown in Table 2 and Figure 1.

### 3.3. Correlation Between BNP Indexes and UCG Indexes

No meaningful correlation was found between BNP levels and LAD, LVEDD, LVMI, and NYHA classification (*p* > 0.05). In contrast, a positive correlation was identified between BNP levels and LVESD (*r* = 0.342, *p* < 0.05), as well as a negative correlation with LVEF (*r* = −0.415, *p* < 0.05), as illustrated in Table 3.

### 3.4. Specificity, Accuracy, and Sensitivity for Different BNP Cut-Off Values

The specificity, accuracy, and sensitivity of BNP cut-off value of 98.9 were 83.05%, 88.14%, and 91.53%, respectively, which were higher than those of other BNP cut-off values (*p* < 0.05), as shown in Table 4.

### 3.5. Value of Plasma BNP Concentration for the Diagnosis of LHF

**Figure 2:**
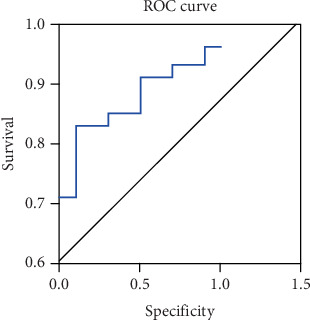
Value of plasma BNP concentration for the diagnosis of left heart failure.

The accuracy of plasma BNP concentration in disease diagnosis was represented by the area under the ROC curve (AUC), with the confidence intervals (CIs) ranging from 0.924 to 0.991, as illustrated in Figure 2.

### 3.6. Analysis of Cardiac Indexes and General Data Characteristics of Different Prognosis Groups in the LHF Group

**Figure 3:**
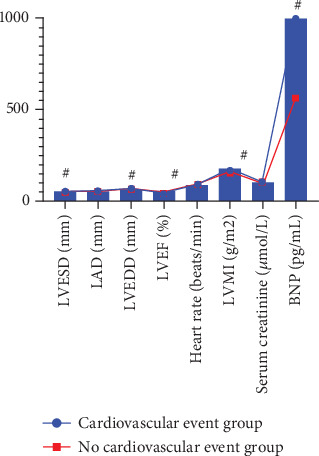
Characteristics of the cardiac indexes and general information of the different prognostic groups in the LHF group. Note: ^#^*p* < 0.05 compared with the group with cardiovascular events.

The results of the follow-up indicated that among the 59 patients, there were 48 individuals who did not experience cardiovascular events, while 11 patients did. This latter group included seven individuals who had a worsening of cardiac function that necessitated readmission and four patients who succumbed to cardiogenic death. In both cohorts, the indexes of LAD, heart rate, and blood creatinine did not demonstrate statistically significant differences (*p* > 0.05). However, patients who suffered cardiovascular events exhibited higher measurements of LVESD, LVEDD, LVMI, and BNP compared to those without such events (*p* < 0.05). Additionally, the LVEF values in patients experiencing cardiovascular events were significantly lower than in the nonevent group (*p* < 0.05). These findings are summarized in Table 5 and illustrated in Figure 3.

### 3.7. ROC Curve for the Prediction of Adverse Events by Plasma BNP

**Figure 4:**
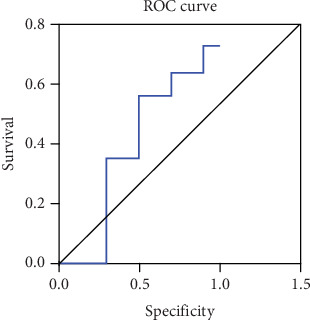
ROC curve of plasma BNP for adverse event prediction.

The ROC curve's area for forecasting negative cardiovascular events using plasma BNP over a 3-month period was measured at 0.738, as illustrated in Figure 4.

The specificity, accuracy, and sensitivity of the BNP cut-off value of 98.9 were 83.05%, 88.14%, and 91.53%, respectively, which were higher than those of other BNP cut-off values (*p* < 0.05). Additionally, the optimal BNP threshold was determined using ROC curve analysis. The AUC for BNP was found to be 0.924–0.991, indicating strong diagnostic performance. The ROC curve further validated the BNP cut-off value of 98.9, supporting its clinical applicability in predicting adverse cardiovascular events, as shown in Figure 4.

### 3.8. Sensitivity Analysis and Validation Outcomes

**Figure 5:**
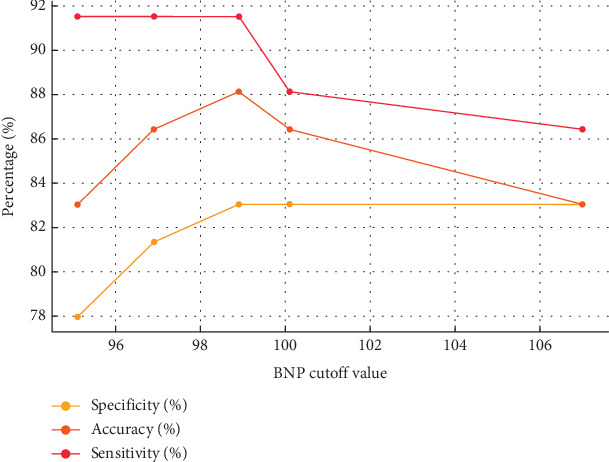
Sensitivity analysis and validation of BNP cut-off values.

The sensitivity analysis showed that varying the BNP cut-off points slightly altered the sensitivity and specificity values, but the overall diagnostic accuracy remained high. Furthermore, the bootstrap validation confirmed the stability of our results, with a minimal variation in the BNP cut-off value's predictive performance across different patient subsets, as shown in Figure 5.

## 4. Discussion

Plasma BNP is a peptide secreted by the ventricles, has a long half-life in the blood, and is significantly correlated with the patient's cardiac function, currently attracting widespread attention from clinical scholars [7]. A study confirmed that transmural pressure loading, cardiomyocyte traction, all cause an increase in the plasma BNP index, whose main synthetic form is an amino acid precursor protein, which is modified intracellularly, and the release of this factor has an impact on gene expression [8]. Plasma BNP is highly expressed in the pathophysiology of LHF disease, and as the disease progresses, BNP is produced by cardiomyocytes, which promote natriuresis and vasodilatation, so plasma BNP indicators can be used as important indicators for disease and prognosis and will provide good guidance for disease treatment, which has good application prospects in disease diagnosis [9, 10]. This research indicates that levels of BNP are significantly increased in individuals suffering from chronic LHF and show a notable correlation with indexes such as LVESD and LVEF. Nonetheless, the diagnostic significance of BNP can differ based on the patient's phenotype. For instance, patients who are obese and have T2DM might display BNP levels that are lower than anticipated, whereas those with CKD and atrial fibrillation could present higher levels of BNP. Therefore, in clinical practice, it is crucial to adjust the BNP reference values and diagnostic criteria according to the specific characteristics of the patient to improve diagnostic accuracy across different HF phenotypes. Additional investigations are required to assess the diagnostic effectiveness of BNP within these specific subgroups, aiming to validate its role as a diagnostic instrument. Alongside the primary evaluation, a sensitivity analysis was performed to determine the consistency of our results. We examined how varying BNP cut-off thresholds, patient demographics, and clinical situations influenced the diagnostic and prognostic effectiveness of BNP. The sensitivity analysis demonstrated that our findings were stable across different scenarios, highlighting BNP's dependability as a diagnostic and prognostic indicator in chronic LHF. Plasma BNP can serve as a strong predictor for suspected conditions in LHF and possesses significant screening capabilities. Currently, rapid plasma BNP tests are widely used in intensive care units and emergency departments to obtain rapid test results, which can confirm the diagnosis of disease and develop effective treatment measures in a short time [11, 12].

The cardiac natriuretic peptide system becomes active during the initial phases of LHF, with plasma levels of BNP rising swiftly within a brief timeframe. Moreover, because of the many predisposing factors of LHF disease, overdiagnosis or underdiagnosis often occurs, which delays the diagnosis of the disease and increases the rate of misdiagnosis, especially in elderly patients with the disease [13, 14]. In addition, because patients often have cough and weakness, clinical diagnosis is often confused with other similar diseases such as clinical lung infections and bronchial tubes, leading to incorrect application of clinical diuretics and other diuretics, which can induce hypotension and insufficient cardiac output, which can interfere with the prediction of disease [15–17]. In this research, the progression of LHF was assessed through plasma BNP testing. The analysis of the prognosis revealed that some patients experienced negative cardiovascular events. Specifically, there were 59 patients without cardiovascular incidents and 11 who did experience such events, which included seven patients readmitted due to worsening cardiac function and four patients who suffered cardiogenic death. Early detection of BNP indexes is beneficial to determine the status of ventricular function, and timely adjustment of the treatment plan and dose of medication can be made by index detection, which can prolong the time to cardiovascular events [18, 19].

In this study, we analyzed patients with 3-month continuous follow-up and plotted the ROC curve of plasma BNP for adverse event prediction. The specificity, accuracy, and sensitivity were 83.05%, 88.14%, and 91.53%, respectively, at a BNP cut-off value of 98.9. We also used ROC curve analysis to assess the optimal BNP threshold for predicting adverse cardiovascular events. The AUC ranged from 0.924 to 0.991, confirming the high diagnostic performance of BNP. This ROC analysis not only supports the selected cut-off value of 98.9 but also underscores the role of ROC curves in optimizing the diagnostic threshold for BNP in chronic LHF [20–22]. However, BNP levels exhibit variability across different states of cardiac function. In this investigation, the Spearman method was utilized to assess the relationship between BNP indexes and cardiac parameters. The findings indicated that as the cardiac function grade improved, BNP concentrations also rose, demonstrating a positive correlation between the two. Additionally, concentrations of BNP showed a positive correlation with LVESD (*r* = 0.342, *p* < 0.05) and changes in LVEF. Variations in BNP concentrations can lead to alterations in clinical symptoms. Furthermore, testing BNP indexes allows for an accurate evaluation of LHF disease progression and effectively differentiates heart failure from LV insufficiency, aligning with the outcomes of this research [23, 24]. Therefore, it is important to develop clinical treatment measures by detecting BNP index, but clinical diagnosis also needs to be combined with patients' clinical symptoms, and this diagnostic method cannot completely replace other effective diagnostic methods but should complement each other to provide guidance value for disease diagnosis [25, 26].

In this study, we acknowledge that the small sample size may limit the generalizability of the results and the robustness of the statistical analysis. Therefore, future research should consider increasing the sample size to further validate the applicability and reliability of these findings.

## 5. Conclusion

This research indicates that levels of BNP are markedly increased in individuals suffering from chronic LHF and demonstrates a significant relationship with essential cardiac function parameters, including LVESD and LVEF. These results emphasize the potential use of BNP as a crucial biomarker for both diagnosing and predicting outcomes in LHF, thereby facilitating the customization of treatment plans according to BNP levels. Moreover, the investigation highlights the importance of factoring in individual patient characteristics, such as obesity, T2DM, and CKD, when assessing BNP levels for improved diagnostic precision among various heart failure types. Although testing BNP concentrations serves as a strong and dependable resource in clinical settings, it should be used alongside other diagnostic techniques to ensure a thorough assessment of the patient's health status. Further studies with larger cohorts are suggested to corroborate these results and to investigate the applicability of BNP in varied patient demographics.

## Figures and Tables

**Table 1:** Profile of cardiac indexes and characteristics of various general data in two cohorts.

**Indicators**	**LHF group (** **n** = 59**)**	**Healthy control group (** **n** = 59**)**	**χ** ^2^/**t**	**p**
Gender (female/male)	24/35	22/37	2.965	0.085
Age (years)	53.41 ± 5.27	53.42 ± 5.21	0.010	0.992
Diastolic blood pressure (mmHg)	81.26 ± 11.47	78.04 ± 10.31	1.604	0.111
Systolic blood pressure (mmHg)	129.42 ± 9.68	127.54 ± 9.52	66.121	< 0.001
LVESD (mm)	44.06 ± 12.35	29.42 ± 3.38	8.782	< 0.001
LAD (mm)	47.52 ± 5.68	35.12 ± 3.84	13.892	< 0.001
LVEDD (mm)	60.35 ± 3.75	48.25 ± 3.68	17.690	< 0.001
LVEF (%)	49.36 ± 5.24	69.64 ± 7.63	16.829	< 0.001
Heart rate (beats/min)	85.26 ± 5.01	73.51 ± 4.63	13.230	< 0.001
LVMI (g/m^2^)	153.24 ± 17.25	81.63 ± 14.26	24.577	< 0.001
Blood creatinine (*μ*mol/L)	102.36 ± 15.24	84.36 ± 11.27	7.294	< 0.001
CRP (mg/m)	11.26 ± 1.02	7.38 ± 0.79	23.100	< 0.001
BNP (pg/mL)	325.68 ± 14.25	18.36 ± 7.24	147.686	< 0.001
Comorbidities				
Obesity (*n*/%)	9 (32.2%)	8 (13.6%)	8.632	< 0.05
Type 2 diabetes mellitus (*n*/%)	14 (23.7%)	5 (8.5%)	5.956	< 0.05
Chronic kidney disease (*n*/%)	11 (18.6%)	2 (3.4%)	7.903	< 0.05
Atrial fibrillation (*n*/%)	8 (13.6%)	3 (5.1%)	3.124	0.077

**Table 2:** BNP levels at different states of cardiac function.

**Cardiac function classification**	**Number of examples**	**BNP concentration (pg/mL)**
NYHA Class II	19	371.52 ± 14.25
NYHA Class III	13	552.68 ± 15.41^∗^
NYHA Class IV	27	823.45 ± 17.36^∗^^#^

⁣^∗^*p* < 0.05 compared with NYHA Class II; ^#^*p* < 0.05 compared with NYHA Class III.

**Table 3:** Correlation between BNP indexes and cardiac parameters.

**Cardiac function classification**	**Correlation with BNP**	**p**
LVESD	0.342	0.004
LAD	0.005	0.971
LVEDD	0.289	0.017
LVEF	−0.415	< 0.001
LVMI	0.156	0.176
NYHA classification	< 0.001	0.537

**Table 4:** Different BNP cut-off values correspond to specificity, accuracy, and sensitivity.

**BNP cut-off value**	**Specificity**	**Accuracy**	**Sensitivity**
107.0	83.05%	83.05%	86.44%
100.1	83.05%	86.44%	88.14%
98.9	83.05%	88.14%	91.53%
96.9	81.36%	86.44%	91.53%
95.1	77.97%	83.05%	91.53%

**Table 5:** Analysis of the characteristics of the prognostic cardiac indexes and general data of different groups in the LHF group.

**Indicator**	**Group with cardiovascular events (** **n** = 11**)**	**No cardiovascular events group (** **n** = 48**)**	**X** ^2^/**t**	**p**
LVESD (mm)	52.21 ± 7.42	43.21 ± 6.35	4.110	< 0.001
LAD (mm)	47.61 ± 6.24	48.26 ± 5.02	0.370	0.713
LVEDD (mm)	67.02 ± 7.34	59.52 ± 5.86	3.651	< 0.001
LVEF (%)	35.21 ± 6.51	50.34 ± 8.41	5.582	< 0.001
Heart rate (beats/min)	86.52 ± 6.24	86.24 ± 6.41	0.131	0.896
LVMI (g/m^2^)	174.25 ± 10.41	151.36 ± 9.56	7.049	< 0.001
Blood creatinine (*μ*mol/L)	100.15 ± 5.25	101.53 ± 9.36	0.130	0.894
BNP (pg/mL)	995.32 ± 14.24	561.52 ± 8.72	130.907	< 0.001

## Data Availability

The data that support the findings of this study are available from the corresponding author upon reasonable request.
